# Systematic C–C Bond Cleavage in Oligomers via Diels–Alder Reaction on Au(111)

**DOI:** 10.1021/acsnano.5c12424

**Published:** 2025-10-01

**Authors:** Donglin Li, Tatsuhiko Ohto, Tomohiko Nishiuchi, Shino Takeuchi, Yuki Nishide, Hajime Kimizuka, Takashi Kubo, Shigeki Kawai

**Affiliations:** † Center for Basic Research on Materials, National Institute for Materials Science, Tsukuba 305-0047, Japan; ‡ Graduate School of Engineering, 12965 Nagoya University, Nagoya 464-8603, Japan; § Department of Chemistry, Graduate School of Science, 13013 Osaka University, Toyonaka 560-0043, Japan; ∥ Graduate School of Pure and Applied Sciences, University of Tsukuba, Tsukuba 305-8571, Japan

**Keywords:** on-surface synthesis, fragmentation, Diels−Alder reaction, scanning tunneling microscopy, density functional theory

## Abstract

On-surface synthesis became a powerful strategy to synthesize extended nanocarbon materials, such as oligomers and graphene nanoribbons, via C–C bond formation between small precursor molecules. However, the reverse reaction, namely, C–C bond cleavage, remains challenging due to the high activation barrier. Here, we present systematic fragmentation to individual units from tetra­(9-anthryl)­benzene oligomers, which were synthesized by Ullmann-type homocoupling on Au(111). The detailed mechanism of fragmentation was investigated with a combination of scanning tunneling microscopy and density functional theory calculations. We found that the Diels–Alder reaction between anthracene groups in the unit significantly lowers the activation barrier to cleave the C–C bond between the units in the oligomer. Our findings may offer an approach to disassemble oligomers in a controlled manner.

## Graphical abstract

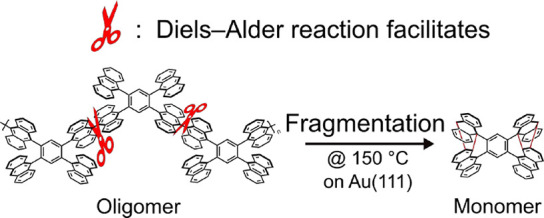

Since Staudinger synthesized long molecular chains connected with covalent bonds between monomeric repeat units in 1922, polymer chemistry has been extensively investigated.^1^ Various functions in extended polymers, such as water impermeability, corrosion resistance, high strength-to-weight ratios, and so on, have been realized.^2–7^ The pursuit of controllable degradation has also emerged as a pivotal avenue, ultimately aiming for enhancing sustainability and enabling diverse applications in fields ranging from biomedicine to environmental engineering, such as drug release, recycling, or waste management.^8–12^ One common approach to achieve controlled degradation is to introduce specific chemical compositions and structures into polymers, which can be decomposed under certain conditions. For instance, acetal, amide groups, o-nitrobenzyl groups, and ester bonds in polymers are degraded in response to environmental factors such as pH, temperature, light, or the presence of enzymes, respectively.^13–17^ To increase the variety of polymer fragmentation, it is important to investigate the mechanism at the atomic scale.

In recent years, the development of on-surface synthesis has facilitated interdisciplinary research between wet and on-surface chemistry, which has led to notable advancements in realizing functional molecular nanoarchitectures.^18–21^ In the reaction, designer precursor molecules are deposited onto surfaces under ultrahigh vacuum conditions and subsequently connected via thermally and optically activated chemical transformation.^22^ Atomic force microscopy (AFM) and scanning tunneling microscopy (STM) with a CO-terminated tip became an essential technique because structures of products can be readily identified by bond-resolved imaging.^23,24^ The direct visualization of inner structures of molecules allows the investigation of reaction pathways, leading to the rapid development of on-surface synthesis. So far, various on-surface reactions have been developed to fabricate extended nanocarbon materials.^25–31^ Among them, Ullmann-type coupling—one of the most utilized reactions—has been employed to synthesize various oligomers^32–34^ and two-dimensional covalent organic structures.^35–38^ Such products offer a playground to investigate mechanical, electronic, and magnetic properties at the atomic scale with STM. The reverse process, namely fragmentation, has also been observed in some systems—for instance, the loss of methyl groups during cyclodehydrogenation reactions^39^ and the fragmentation of individual molecules.^40^ These examples provided valuable insights into the on-surface chemistry. However, the fragmentation of oligomers on surfaces remains challenging due to the high activation barrier of C–C bond cleavage, which is critical for disassembling oligomers in a controlled manner.

Here, we use 1,2,4,5-tetra­(10-bromo-9-anthryl)­benzene (**1**) to study the fragmentation. Annealing **1** on the Au(111) surface at 100 °C leads to the synthesis of tetra­(anthryl)­benzene oligomers through the Ullmann-type homocoupling at 100 °C (Scheme 1). Subsequently, **1** oligomer undergoes a sequential transformation to form compound **4**. In the first step of fragmentation, an intramolecular Diels–Alder reaction occurs between adjacent sterically hindered anthracene units. Subsequently, partial cleavage of the newly formed C–C bond leads to the generation of the tetraradical intermediate **A**. Due to its high reactivity, intermediate **A** undergoes hydrogen abstraction to yield compound **3**. Since **3** contains a helicene structure, further cyclodehydrogenation proceeds upon heating, affording compound **4**. The **4** derivatives can also be synthesized through a similar reaction path while dissociating one or two phenyl groups during annealing (Scheme S1). The presence of **2**, which was synthesized through the fragmentation of the **1** oligomer, was confirmed via the analysis of the reaction pathway based on the observed planar compounds with a combination of bond-resolved STM and density functional theory (DFT) calculations.

**Scheme 1:**
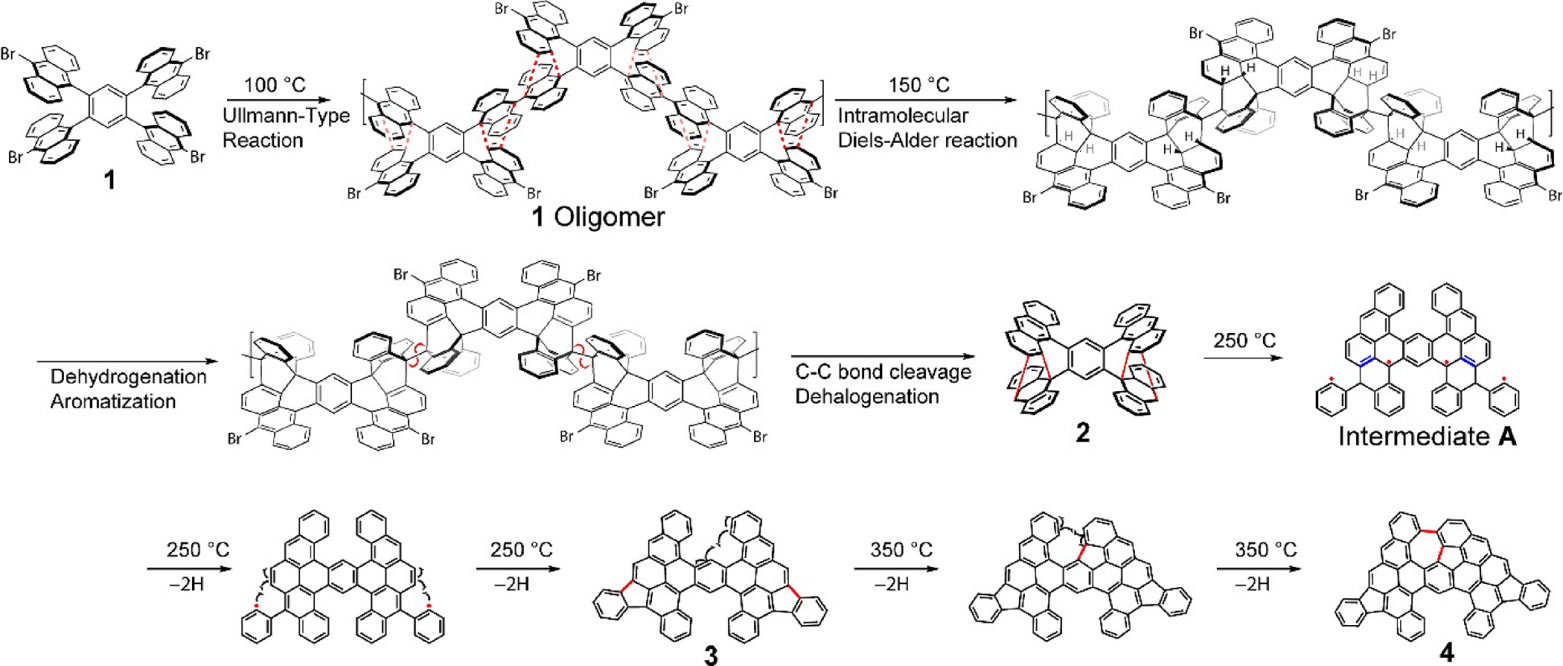
A Series of On-Surface Reactions Illustrating the Structural Evolution^a^

a Oligomerization occurs at 100 °C via Ullmann-type coupling, forming **1** oligomer. Upon annealing at 150 °C, fragmentation takes place through an intramolecular Diels–Alder reaction, leading to the formation of **2**. Subsequent annealing at 250 and 350 °C induces planarization, where **2** is converted into the conjugated **3** and **4**.

## Results and Discussion

Upon depositing **1** on the Au(111) surface kept at room temperature, an extended self-assembled structure was formed (Figure 1A). Since the gold herringbone pattern is seen on the molecular island, the interaction between the molecule and the substrate was relatively weak, namely physisorption. Further, the presence of the herringbone structure indicates the absence of debromination, and so the molecule was intact. The close-up view of the molecular island shows chevron-like chain structures (Figure 1B). The large height of the island (361 pm) indicates that the molecule is nonplanar, as expected from the chemical structure of **1**. To investigate the assembly, individual molecules were removed from the island by scanning the tip at a closer tip–sample distance along the trajectories indicated by the arrows. We found the separated molecule in a dumbbell shape, which is in agreement with the simulated STM image with a DFT relaxed structure on the Au(111) surface (Figure 1C). Assigning the shape of the missing molecules in the island, the position of **1** was determined as indicated by black contours in Figure 1b. Thus, the dark line in the molecular island corresponds to the central benzene in compound **1**. The molecules were most probably condensed by the Br···Br–C halogen bonding and Br···H–C hydrogen bonding.

**Figure 1:**
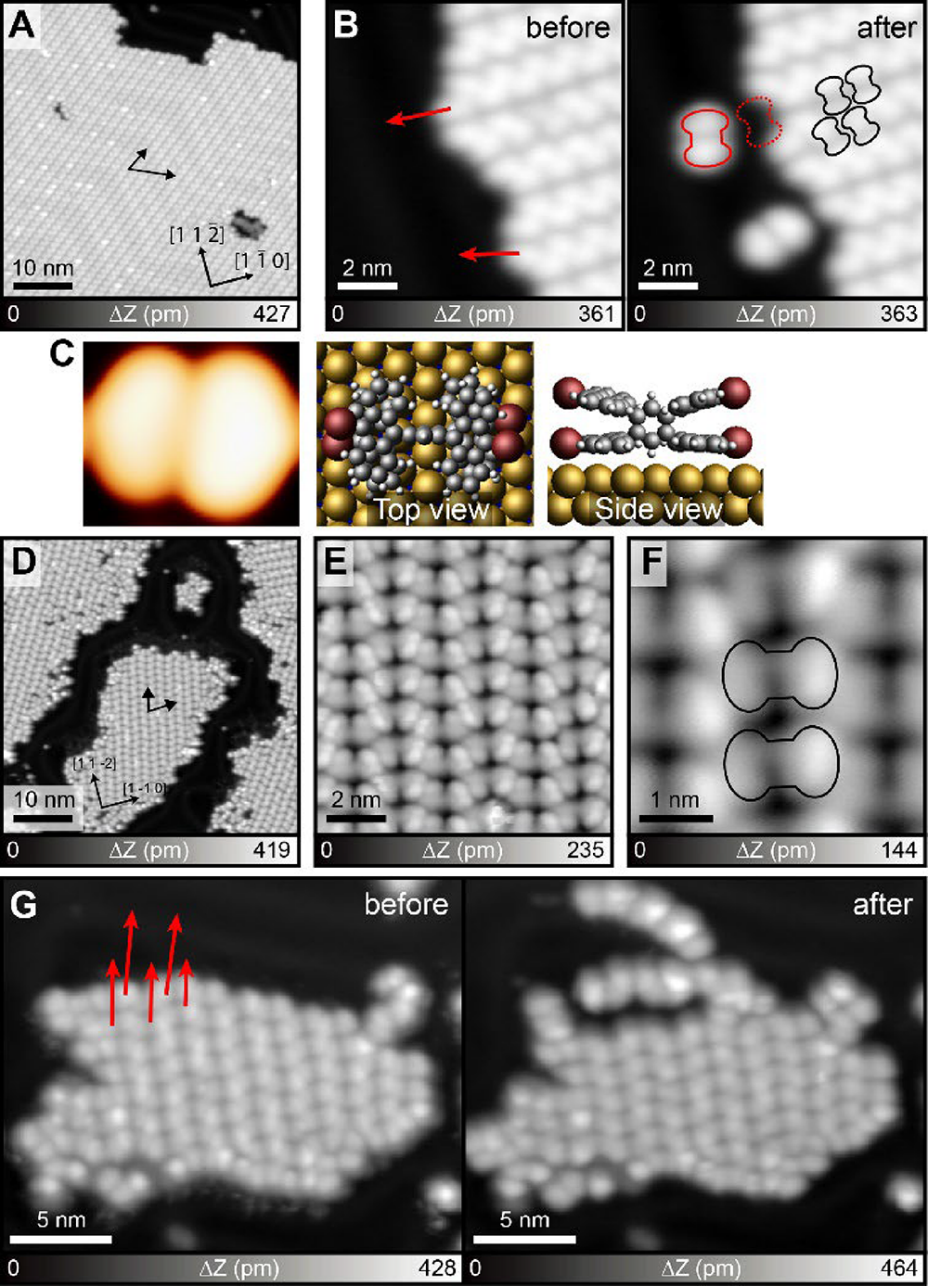
On-surface synthesis of oligomers. (A) Large-scale STM topography taken after deposition of **1** on Au(111). (B) Close-up views before and after the manipulation of individual molecules. The red arrows indicate the direction of the tip movement during the manipulation. The red solid and dotted contours indicate the manipulated molecules and the resulting vacancies, respectively. The black contours indicate the individual molecules in the self-assembled island. (C) Simulated STM image on the left. Top and side views of the DFT relaxed structure in the middle and right. (D) Large-scale and (E, F) close-up STM topographies taken after annealing at 100 °C. The molecules are labeled by black contours. (G) STM topographies taken before and after several manipulations. Two oligomers were separated from the molecular island. Measurement parameters: Sample bias voltage *V* = 800 mV and *I* = 2 pA in (A, B), and *V* = 500 mV and *I* = 2 pA in (D–G).

After annealing the sample at 100 °C, we found that the growth direction of the molecular unit in the island changed, as indicated by black arrows in Figure 1D. To investigate the structure, a close-up view image was taken on the island (Figure 1E). We could identify the molecular site by assigning the dumbbell pattern (Figure 1F), yet the corrugated structure prevented detailed structural analysis of whether the molecular island corresponds to a two-dimensional covalent network or self-assembled one-dimensional oligomers. Thus, we attempted to modify the island by tip-induced manipulation (Figures 1G and S1). Since chains were separated, we concluded that the molecular island was composed of self-assembled one-dimensional oligomers. The oligomer was detached by tip manipulation while remaining structurally intact, suggesting covalent bonding between the molecular units.^41–43^ Therefore, the **1** oligomer was formed with an abundance of 69% which was obtained by counting the number of units (Figure S2). Since the herringbone pattern on the molecular island disappeared after the annealing, the dissociated bromines participated in the oligomer self-assembly and condensed them by the hydrogen bonding. In fact, the bromine atoms appeared beside the oligomers after manipulation (Figure S3), further supporting the formation of polymers via Ullmann-type coupling. Notably, the different appearance of the extracted polymer compared to the contour observed within the island arises from the intrinsic flexibility of the molecular backbone. The C–C single bonds between the anthracene groups as well as between the anthracene and phenyl groups allow conformational variations, and the three-dimensional configuration of the molecular units further contributes to bending during manipulation. We deduce that only two aryl bromine groups in **1** in close proximity to the surface were reacted at 100 °C due to the gold catalysis (Scheme 1). In contrast, the other two aryl bromine groups positioned farther away from the substrate should remain intact, which can be confirmed by employing Br-functionalized tips and tip-induced debromination (Figure S4).

To induce the reaction of the remaining C–Br bonds in the oligomer, the sample was annealed at 150 °C. Surprisingly, we found that all oligomers were transformed into small molecules (Figure 2A) with 98% abundance. It is known that even at a higher temperature of 400 °C, the C–C single bond in oligomers usually remains intact.^44^ Rather, π-extension is often caused by thermal fusion at the edge.^45^ The inset of Figure 2A shows that the molecule dissociated from the oligomer by annealing is smaller than **1** detached from the molecular island by the tip. The line profile of the dissociated molecule, taken along its longitudinal axis, clearly differs from that of compound **1**. It exhibits reduced symmetry, as shown in Figure S5. Apparently, the two compounds are not the same. The Diels–Alder reaction has been demonstrated to occur on surfaces, and its reactivity with anthracene groups is also well-established.^46–50^ Therefore, it is reasonable that the Diels–Alder reaction takes place between adjacent anthracene units within **1**. To verify the hypothesis, we optimized the structure of **2** on the Au(111) surface using DFT calculations and subsequently simulated the STM image. The contrast of the simulated image shows good agreement with the experimental data (Figure 2B), supporting that the structure of the dissociated molecule should correspond to **2** (Figure 2C). Notably, a mirror-symmetric configuration of **2** was used here, while the possibility of a helical **2** is discussed in Scheme S2. In addition, the STS results of **1** and **2** (Figure S6) reveal distinct electronic properties, further indicating that **2** is a new species formed via the Diels–Alder reaction. We assumed that the intramolecular Diels–Alder reaction also relates to the unusual fragmentation because the annealing temperature of 150 °C is not high enough to overcome the activation barrier of the C–C bond cleavage between the units on Au(111). It should be noted that retro-Diels–Alder reactions may occur on the surface so that oligomer **1** and the intramolecular DA product coexist in thermal equilibrium. However, once the intramolecular DA product undergoes dehydrogenation and subsequent aromatization to form **2**, the system cannot revert to the original oligomer **1**. To investigate the structure of the dissociated molecule experimentally, we induced planarization by annealing at 250 °C, and three main isolated products were formed that together accounted for ∼35% of the total molecular structures (Figure 2D). The remaining products consisted of various minority nonplanar structures and fused oligomers. The close-up view shows an example of the trapezoidal-shaped molecule (inset of Figure 2D). We attempted to resolve the inner structure by bond-resolved STM with a CO-terminated tip, but found that the accidental movement of the molecule prevented high-resolution imaging. The low diffusion barrier most probably resulted from the nonplanar molecule (Figure S7). The structure of the molecule was thus investigated by considering the transformation to the quasiplanar compound from compound **2** (Scheme 1). To validate the analysis, we conducted DFT calculations and obtained an agreement between the simulated STM and experimental data (Figures 2E and S8). Thus, the trapezoidally shaped molecule should correspond to **3** (Figure 2F). To planarize the compound completely, the sample was further annealed at 350 °C (Figure 2G). The close-up view of the STM topography shows that the trapezoidal structure became less mirror-symmetric (inset of Figure 2G). The corresponding bond-resolved STM image revealed the inner structure, named **4** (Figure 2H). We found that the formation of the central five- and seven-membered rings accounts for the observed asymmetry. This compound can be obtained from **3** through cyclodehydrogenation, as indicated by the red lines in Figure 2I. This provides direct evidence that the Diels–Alder reaction occurred and that compound **2** was formed. We also observed smaller products (**3′**, **3″**) after annealing at 250 °C (Figure S9). Similar to **3**, the chemical structures could not be identified by bond-resolved imaging due to accidental manipulations. Thus, we planarized them by annealing at 350 °C. The bond-resolved images of the final products indicate that the intermediates of **3′** and **3″** correspond to **3** with one or two phenyl groups missing (Figure S10). Statistical analysis of each reaction step is provided in Figure S11. Taken together, the intramolecular Diels–Alder reaction induced unusual fragmentation of **1** oligomers. It is worth noting that this fragmentation also occurs on the Ag(111) surface (Figure S12).

**Figure 2:**
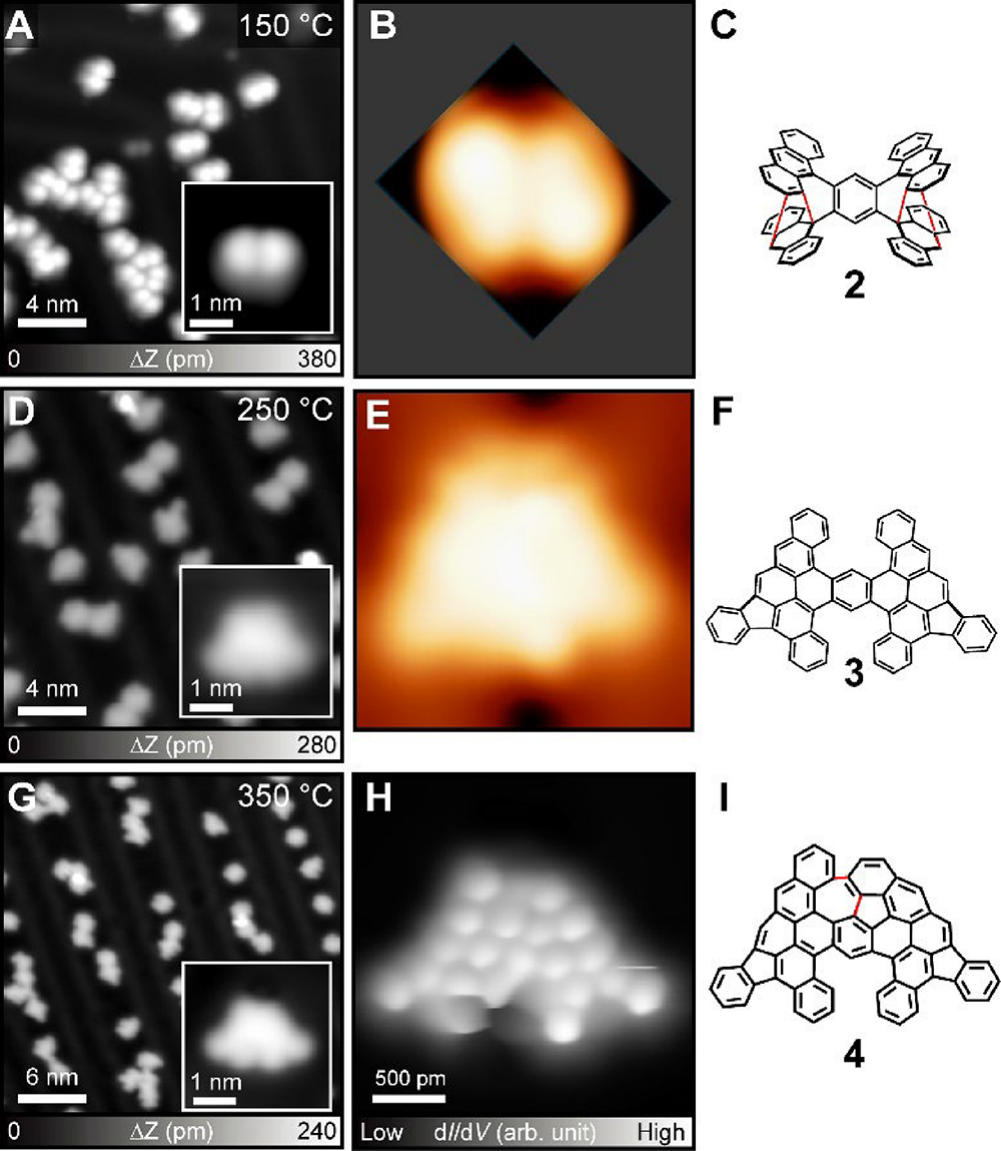
Structural transformation by annealing at different temperatures. (A) Large-scale STM topography taken after annealing the sample at 150 °C. The inset shows the close-up view. (B) Simulated STM image and (C) chemical structure of **2**. (D) Large-scale STM topography taken after annealing at 250 °C, and a close-up view is shown in the inset. (E) Simulated STM image and (F) chemical structure of **3**. (G) Large-scale STM topography taken after annealing at 350 °C, and a close-up view is shown in the inset. (H) Corresponding bond-resolved STM image and (I) chemical structure of **4**. Measurement parameters: *V* = 0.5 V and *I* = 2 pA in (A). *V* = 0.2 V and *I* = 5 pA in (D). *V* = 0.2 V and *I* = 10 pA in (g). *V* = 1 mV in (H).

To verify the intramolecular Diels–Alder reaction, we conducted a complementary experiment with 1,2,4,5-tetra­(9-anthryl)­benzene (**1′**),^51^ in which bromine atoms of **1** were replaced by hydrogen atoms. Depositing **1′** on Au(111) kept at room temperature resulted in the formation of small molecular islands (Figure 3A). In contrast to the self-assembled structure of **1** condensed by Br···Br–Br···H interactions, no two-dimensional extension was seen. The low ordered structure should result from the relatively weak C–H···π interaction between the molecules. The comparative STM profiles for compounds **1** vs **1′** show that the bromine atoms lead to a slightly bigger STM topography. In order to induce the intramolecular Diels–Alder reaction, the sample was annealed at 150 °C (Figure 3B). Since the molecular arrangement on the surface and the shape observed in the STM profile (Figure S13) are identical to those of **2**, we conclude that the Diels–Alder reaction also occurred in **1′**. To induce structure transformations, the sample was further annealed at 250 °C and 350 °C (Figure 3C,D). The close-up views show the excellent consistency of the STM topographic contrast between the products from **1** and **1′**, while the molecule in the inset of Figure 3D has a mirror symmetry with that in the inset of Figure 2G (see Figure S14). Thus, the complementary experiment also supports the occurrence of the intramolecular Diels–Alder reaction.

**Figure 3:**
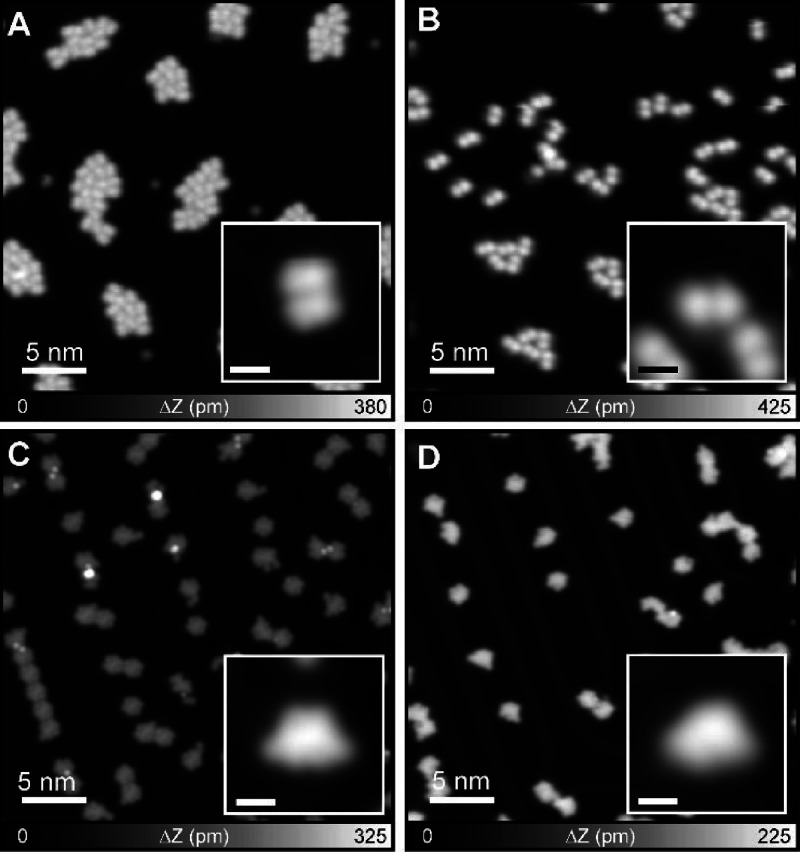
Complementary experiment to verify the intramolecular Diels–Alder reaction with 1,2,4,5-tetra­(9-anthryl)­benzene (**1′**). (A) STM topography of **1′** as-deposited on Au(111). (B) STM topographies obtained after annealing the sample at 150 °C, (C) 250 °C, and (D) 350 °C. Insets show the corresponding close-up views of the molecule. The scale bars in the insets: 1 nm. Measurement parameters: *V* = 0.5 V and *I* = 2 pA in (A,B). *V* = 0.2 V and *I* = 10 pA in (C, D).

To get detailed insight into the fragmentation mechanism, we performed DFT calculations on the Au(111) surface. Given that C–C bond cleavage has a high activation barrier of approximately 83 kcal/mol, simple bond disconnection is unlikely to occur through annealing alone. According to the indication from the experiments, the intramolecular Diels–Alder reaction should play a role in the fragmentation. First, we investigated the energy barrier of the intramolecular Diels–Alder reaction to **2′** from **1′** (Figure 4A). For simplicity, we considered the intramolecular Diels–Alder reaction occurring on only one side. The activation barrier on the surface is significantly reduced compared to that in the gas phase. More importantly, **2′** is thermodynamically more stable than **1′** on the Au surface, although the activation energy barrier between them is relatively small. According to the Boltzmann distribution, the lower energy of **2′** results in a higher population under equilibrium conditions. Therefore, the intramolecular Diels–Alder reaction is promoted on the surface by mild annealing, which is in agreement with the experimental observations. Next, we investigated the role of the intramolecular Diels–Alder reaction in the cleavage of the C–C bond connecting two monomer units (Figure 4B). We calculated dissociation energies of weak **1′** dimer (positioning the center of mass of the benzene rings at approximately 640 pm above the surface, Figure 4C) and strong **1′** dimer (positioning the center of mass of the peripheral rings at approximately 490 pm above the surface, Figure 4D) as −89.94 and −124.53 kcal/mol, respectively. In the case of the **1′** dimer with weak interaction to the substrate (blue curve), the dissociation energy is even higher than that of the typical C–C bond cleavage, which is most probably related to the distortion of flexible peripheral groups caused by adsorption to the substrate. Positioning the C–C bond closer to the surface (green curve), the dissociation energy reduces by 40 kcal/mol, which is still high. Next, we considered the effect of the intramolecular Diels–Alder reaction on the C–C bond cleavage of the **2′** dimer (Figure 4E). Indeed, we obtained a relatively low dissociation energy barrier (red curve). Note that since the potential energy does not reach its maximum, the barrier may be slightly higher at a longer C–C distance. The possibility of the retro Diels–Alder reaction cannot be excluded. Thus, we considered the dehydrated **2′** dimer (Figure S15) and obtained the dissociation energy barrier of 67 kcal/mol (black curve), which can be promoted on the surface. The dehydrogenation barrier of the **2′** dimer was also calculated to be 56 kcal/mol (Figure S16). This is lower than the dissociation energy barrier, suggesting that dehydrogenation could preferentially occur. In addition, it has been demonstrated with relatively planar molecules that the activation barrier in on-surface reaction is generally lowered by both surface-induced strain^52^ and the catalysis of surface gold atoms.^53^ In our case, the 3D structure of **1** gives greater local strain, which should significantly lower the activation barrier for C–C bond cleavage. In contrast to rigid planar molecules, our precursor molecule is flexible so that the structure can be oscillated under annealing conditions. Thus, we expect that the activation barrier can be smaller than that we calculated with the relaxed structure. Besides the local strain, the gold adatoms should also play a role in lowering the activation barrier. Hence, our analysis shed light on the role of Diels–Alder interaction for the oligomer fragmentation.

**Figure 4:**
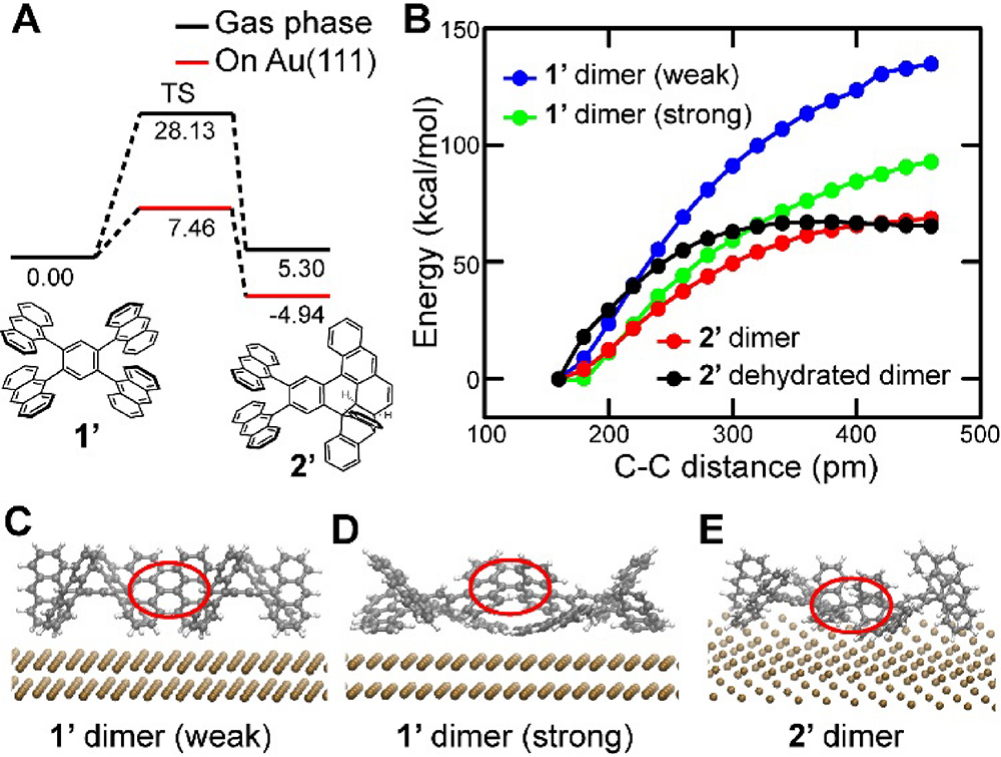
C–C bond cleavage between the intermolecular anthracene units investigated by DFT calculations. (A) Energy landscape of the intramolecular Diels–Alder reaction in **1′**. The unit of energy is kcal/mol. (B) Potential energy surfaces of **1′** and **2′** dimers on the Au(111) surface calculated by pulling the C–C distance. (C) Structures of **1′** dimers with weak and (D) strong π–metal interactions. (E) Structure of **2′** dimers, in which an intramolecular Diels–Alder reaction occurred at the C atoms that are to be dissociated. The C–C bonds between the monomer units are marked with red circles.

## Conclusions

In summary, we demonstrated fragmentation of the oligomer through C–C bond cleavage promoted by the thermally activated intramolecular Diels–Alder reaction between anthracene groups in the unit on the Au(111) surface. The mechanism of the fragmentation process was investigated with a combination of STM and density functional theory calculations. Furthermore, annealing the sample at higher temperatures induced planarization of the dissociated unit, which was investigated by bond-resolved imaging. Combining with the previous on-surface reaction for the π-extended system, this method may provide a more complex carbon nanostructure through systematic connection and fragmentation of molecular units. We believe this study will inspire future development of functional materials that integrate such reactivity, potentially leading to new classes of surface-responsive or degradable polymers.

## Methods

### STM Experiments

A homemade low-temperature STM was utilized in this study under ultrahigh vacuum conditions (*P* < 5 × 10^–10^ mbar) and a low temperature of 4.3 K. The Au(111) substrate underwent cleaning via several cycles of sputtering with Ar^+^ ions and annealing at 430 °C for 15 min. During annealing, the sample temperature was monitored using a thermocouple positioned close to the sample for temperatures below 250 °C, and a combination of a thermocouple and a pyrometer for temperatures above 250 °C. The precursors of **1** and **1′** were prepared in solution following the protocol outlined in the Supporting Information and subsequently evaporated onto the Au(111) surface maintained at room temperature using a standard Knudsen cell (Kentax GmbH). Bond-resolved STM (BR-STM) images were obtained in constant-height mode (V = 1 mV) employing a CO-functionalized tip.^23,54^ A CO molecule was picked up from the surface.^55^ STM imaging was conducted with chemically etched tungsten tips.

### Theoretical Calculations

DFT calculations were performed with the VASP code^56^ using the projected augmented wave (PAW) method.^57^ The Perdew–Burke–Ernzerhof (PBE) functional^58^ was employed as the exchange-correlation functional. The van der Waals correction was included via the Grimme’s D3 method with the Becke-Johnson damping variant.^59^ We employed slab models consisting of molecules, two layers of Au(111) surface, and an approximately 20 Å-thick vacuum layer. The plane wave energy cutoff was set to 400 eV. The gold atoms were fixed at the experimental positions during structural optimization. The local density of states was employed for drawing STM images after applying a Gaussian filter function.

The reaction pathways shown in Figure 4 in the main text were calculated as follows. The nudged elastic band (NEB) method was employed for estimating the activation barrier of the intramolecular Diels–Alder reaction between **1′** and **2′** (Figure 4A). Three intermediate states were employed as images for the NEB calculations. The spring constant between the images was set to 5.0 eV/Å^2^. To calculate potential energy surfaces of C–C bond cleavage (Figure 4B), we first optimized the molecular structures of **1′** dimer (weak p-metal interaction), **1′** dimer (strong p-metal interaction), **2′** dimer, and **2′** dehydrated dimer on the Au(111) surface. All the atoms in molecules were optimized while the positions of Au atoms were fixed. Because the C–C bond cleavage is uphill and the reaction coordinate is long, we gradually elongated the C–C bond connecting two monomer units. The *x* and *y* coordinates of two carbon atoms were fixed, and other degrees of freedom in the dimer were optimized to compute the total energies. The relative energies of the stable dimer structures were plotted against the C–C distance connecting the monomer units.

## Supplementary Material



## Notes

Synthesis of 1,2,4,5-tetra­(10-bromo-9-anthryl), Structural evolution from an intermediate, A series of STM topography during tip-induce manipulation, Reaction yield at each step of fragmentation process, Dissociated bromine atom appeared after manipulation, Debromination process in the oligomer, Close-up view STM images of the monomers, A series of On-surface reactions of helical **1**, Electronic properties of **1**, **2**, **1′**, STM image of **3** and corresponding BR-STM images, Optimized structure of **3**, Close-up views of **3**, **3′** and **3″**, STM topography of planar products, Statics of the outcome products, Formation and fragmentation of the oligomers on Ag(111), Comparative STM profiles for **1** vs **1′** and **2** vs. **2′**, STM images of **1** and **1′**, Structures of **2′** dimer and dehydrated **2′** dimer, Dehydrogenation of **2′** investigated by DFT calculations (PDF)
