# Supplementary material for: Systematic C–C Bond Cleavage in Oligomers via Diels–Alder Reaction on Au(111)
Source: ACS Nano. 2025 Oct 1;19(40):35825–32. doi: 10.1021/acsnano.5c12424 (PMC12530030; doi:10.1021/acsnano.5c12424)
Supplement: Supplementary file 1 [file nn5c12424_si_001.pdf]

Supporting Information

for

Systematic C-C Bond Cleavage in Oligomers via Diels-Alder Reaction on Au(111)

Donglin Li[a], Tatsuhiko Ohto[b], Tomohiko Nishiuchi*[c], Shino Takeuchi[c], Yuki Nishide[b], Hajime Kimizuka[b], Takashi Kubo [c], Shigeki Kawai*[a],[d]*

[a] Dr. D. Li, Prof. Dr. S. Kawai, Center for Basic Research on Materials, National Institute for Materials Science, Tsukuba 305-0047 (Japan).

E-mail: KAWAI.Shigeki@nims.go.jp

[b] Prof. Dr. T. Ohto, Y. Nishide, Prof. Dr. H. Kimizuka, Graduate School of Engineering, Nagoya University, Nagoya 464-8603 (Japan).

E-mail: ohto@nagoya-u.jp

[c] Prof. Dr. T. Nishiuchi, S. Takeuchi, Prof. Dr. T. Kubo, Department of Chemistry, Graduate School of Science, Osaka University, Toyonaka 560-0043 (Japan).

E-mail: nishiuchit13@chem.sci.osaka-u.ac.jp

[d] Prof. Dr. S. Kawai, Graduate School of Pure and Applied Sciences, University of Tsukuba, Tsukuba 305-8571 (Japan).

Synthesis of 1,2,4,5-tetra(10-bromo-9-anthryl)benzene 1

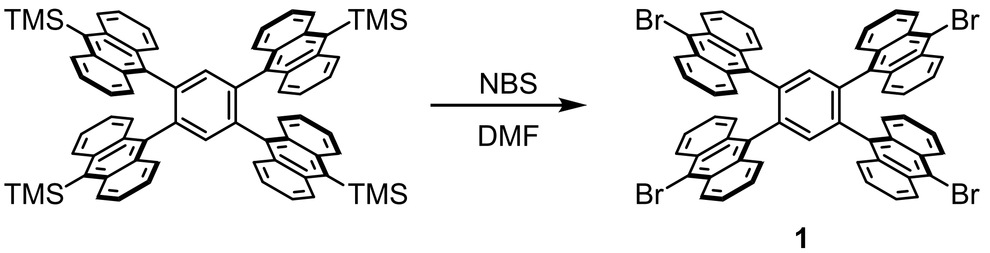

To a solution of 1,2,4,5-tetra(10-trimethylsilyl-9-anthryl)benzene[S1] (54 mg, 0.050 mmol) in DMF (3 ml) was added NBS (45 mg, 0.25 mmol) at room temperature. After stirring for 1 day, a yellow precipitate was collected by filtration and rinsed by dichloromethane. After dried the yellow precipitate under vacuum, 40 mg (0.037 mmol, 73%) of compound **1** was obtained. Mp: >300 °C. MS(APCI): *m*/*z* 1099 [(*M*+H)$^+$]. ^{1}H NMR (700 MHz, $CDCl_3$) δ 9.61 (d, *J* = 8.8 Hz, 8H), 9.54 d, *J* = 8.8 Hz, 8H), 9.46 (s, 2H), 8.68 (m, 8H), 8.60 (m, 8H).; ^{13}C NMR (175 MHz, $CDCl_3$) δ 140.61, 140.10, 136.22, 131.91, 130.98, 129.03, 128.71, 128.11, 126.79, 124.73.

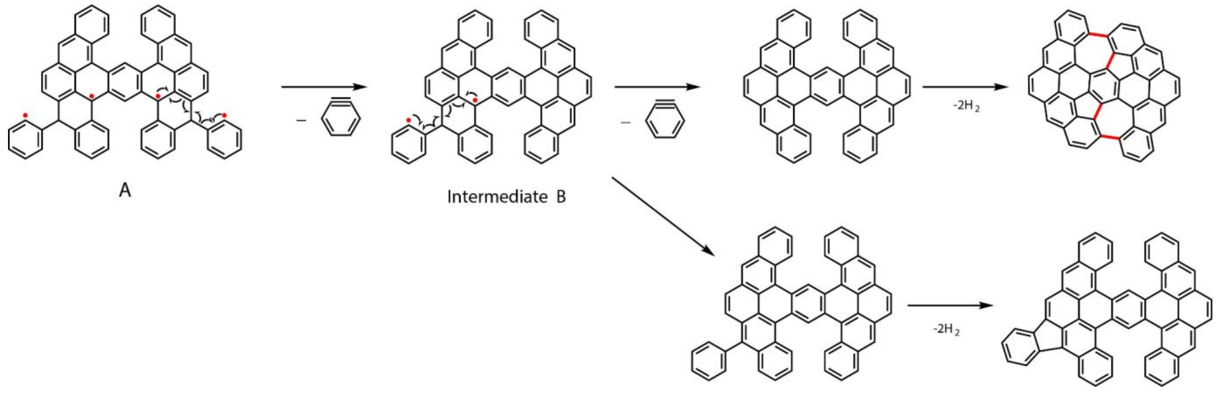

Scheme S1. A series of on-surface reactions illustrating the structural evolution from intermediate **A** to **4** derivatives.

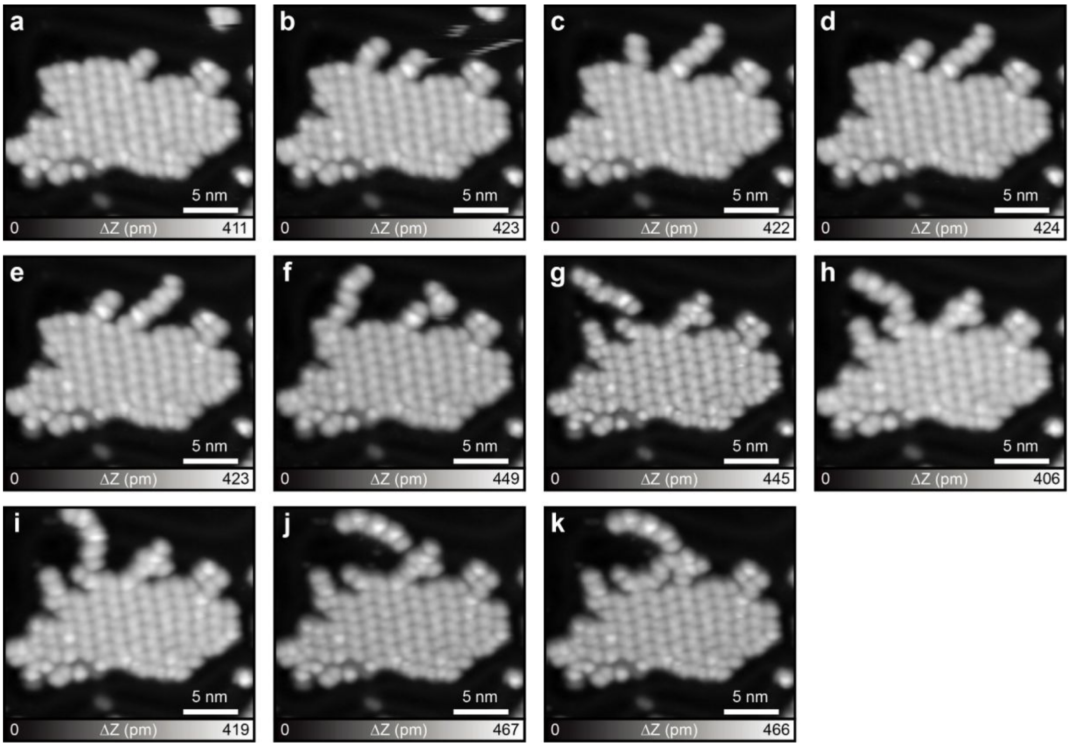

Figure S1. (a-k) A series of STM topographies during tip-induced manipulation, showing that the island is composed of polymer chains. Sample bias voltage V = 500 mV and tunneling current I = 2 pA.

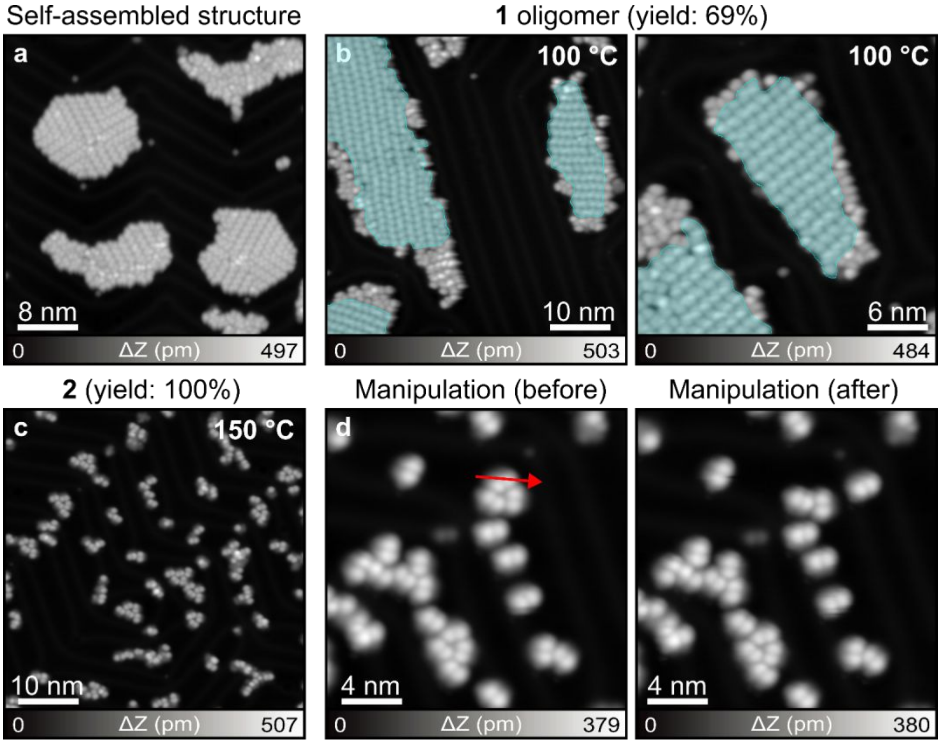

Figure S2. Reaction yield at each step of the fragmentation process. (a) STM image of the self-assembled structure of **1**. (b) STM image of **1** oligomer after annealing the sample at 100 °C. The light blue marked areas indicate **1** oligomer. (c) Large-scale STM image of **2** after annealing the sample at 150 °C. (d) Close-up views before and after manipulation of **2** monomer. The red arrow indicates the direction of the tip movement during manipulation. Measurement parameters: V = 200 mV and I = 10 pA in (a, b, c, d).

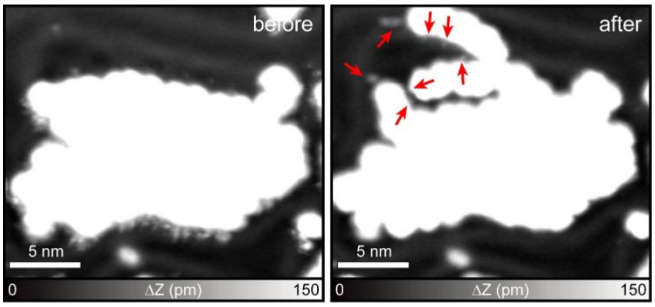

Figure S3. Dissociated bromine atoms appeared after manipulation. The images are the same as those in Figure 1g of the main text. The ΔZ scale was set to 150 pm for better visibility of the dissociated bromine atoms on the surface. The red arrows indicated the bromine atoms.

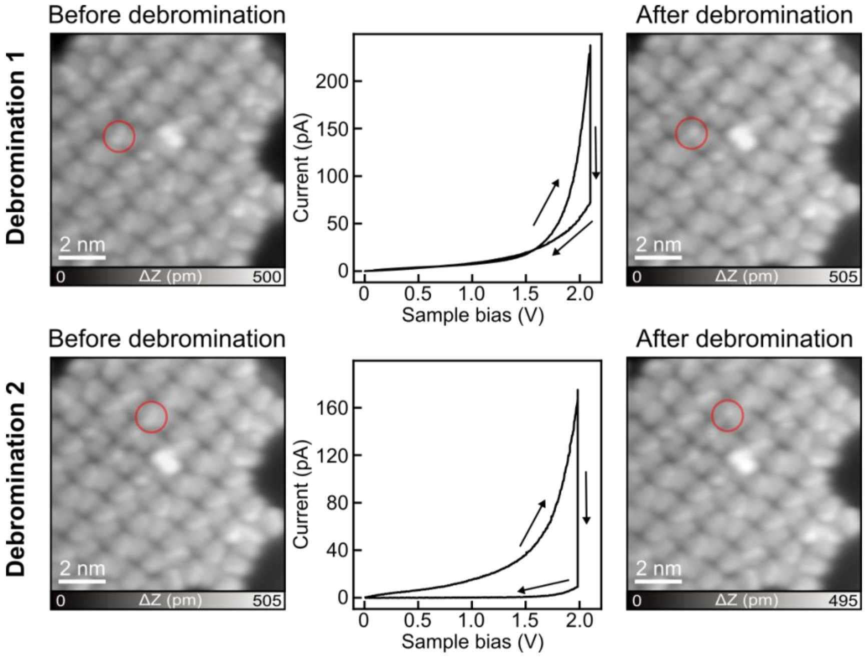

Figure S4. Two typical debromination processes demonstrate the presence of Br atoms in the oligomers. STM images were acquired using a Br-functionalized tip, where Br atoms appear as small bright dots. Red circles mark the debromination sites. After debromination, the two Br atoms highlighted in red circles are reduced to a single Br atom. Measurement parameters: Sample bias voltage $V = 200$ mV and $I = 10$ pA.

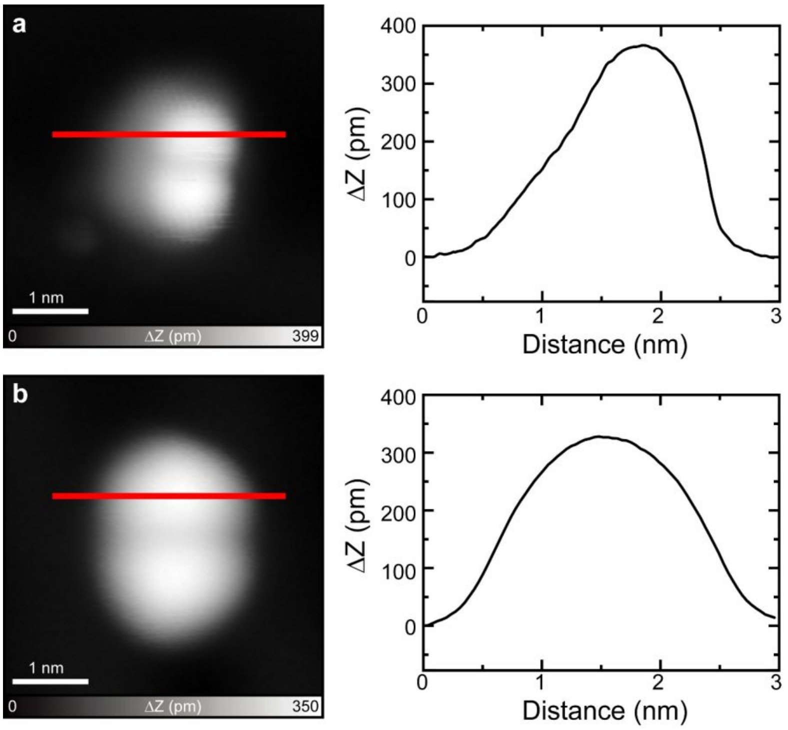

Figure S5. Close-up view STM images of (a) the monomer obtained after annealing the sample at 150 °C and (b) the monomer in the self-assembled structure (left), along with the corresponding line profile along red lines (right). Measurement parameters: (a) $V = 0.8$ V and $I = 2$ pA in (a). $V = 1$ V and $I = 2$ pA in (b).

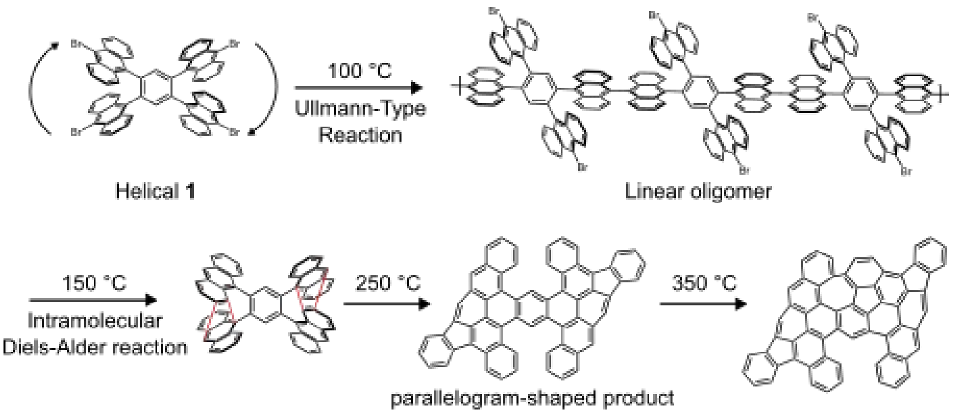

Scheme S2. A series of on-surface reactions illustrating the structural evolution of helical **1**. The formation of helical **2** would require precursor **1** to adopt a helical configuration. In this case, annealing to 100 °C would be expected to yield linear oligomers, and subsequent annealing to 250 °C and 350 °C for planarization should produce parallelogram-shaped products. However, such structures were not observed in our experiments.

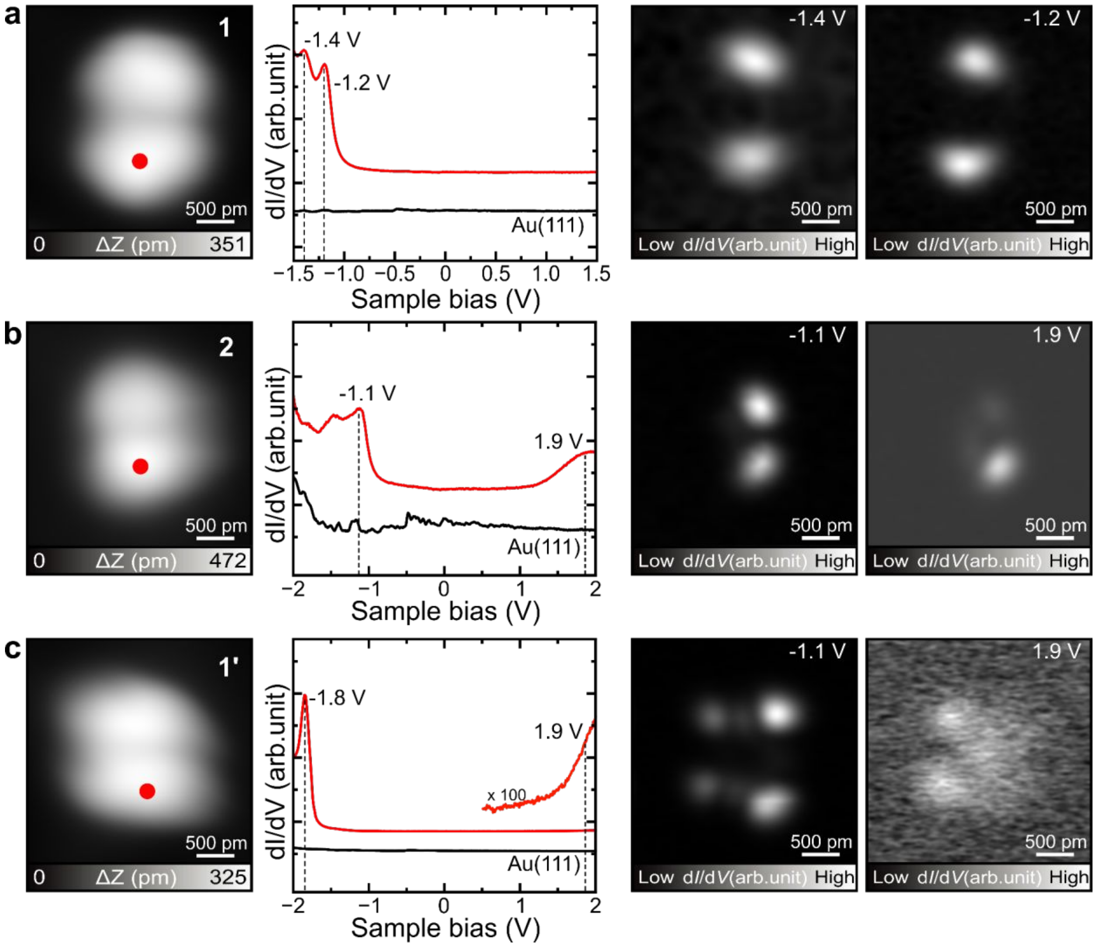

Figure S6. Electronic properties of **1**, **2**, and **1'**. (a) STM topography of **1** displaying site indicated by red dot, where the dI/dV spectrum was obtained. The dark curve represents the spectrum taken on the bare Au(111) surface for reference. The peaks at −1.4 V and −1.2 V correspond to positive ionization resonance (PIR) and PIR-1, respectively. The absence of negative ionization resonance (NIR) is attributed to debromination triggered by the application of a higher voltage. There is no spatial resolution in the dI/dV maps due to the three-dimensional structure. (b) dI/dV spectrum and dI/dV maps of **2**. The peaks at −1.1 V and 1.9 V correspond to PIR and NIR, respectively. (c) The dI/dV spectrum and dI/dV maps of **1'**. The peaks at −1.8 V and 1.9 V correspond to PIR and NIR, respectively. Measurement parameters: STM topography: I = 2 pA and V = 500 mV. dI/dV spectra: I = 50 pA, V = 1 V, lock-in parameters V_{ac} = 10 mV. Constant height dI/dV maps: V_{ac} = 10 mV.

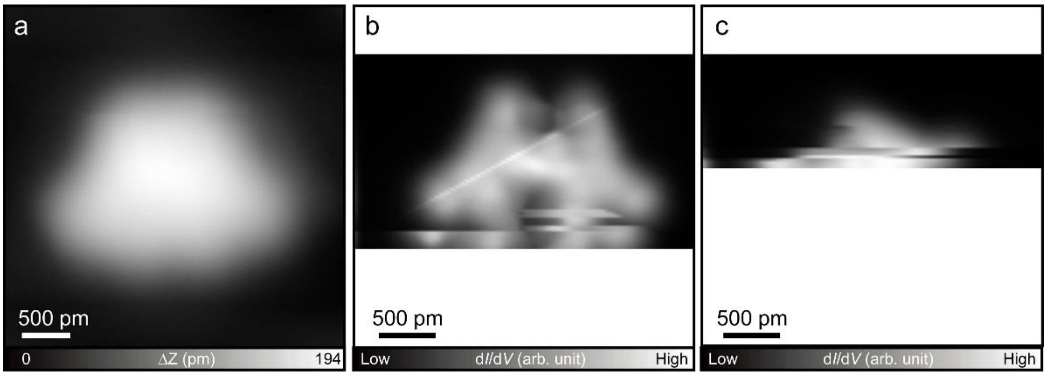

Figure S7. (a) STM image of **3** and (b,c) corresponding BR-STM images. The tip-sample distance of (c) was 5 pm closer than that of (b). The slow scan direction is to the bottom from the top. In both cases, the molecules were accidentally manipulated by the scanning tip. Measurement parameters: $V = 200$ mV and $I = 5$ pA in (a). $V = 1$ mV in (b,c).

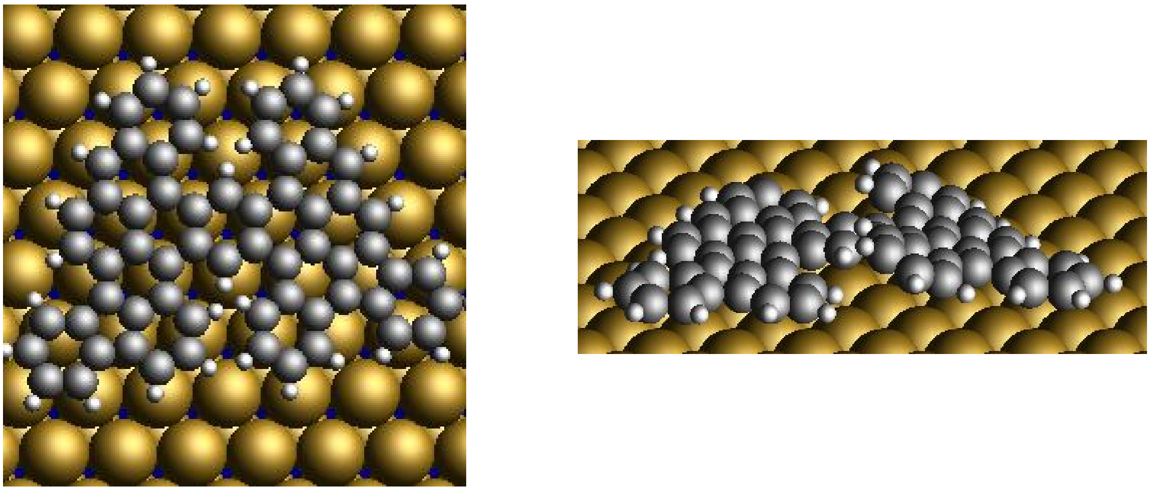

Figure S8. Optimized structure of **3** on the Au(111) surface with DFT calculations.

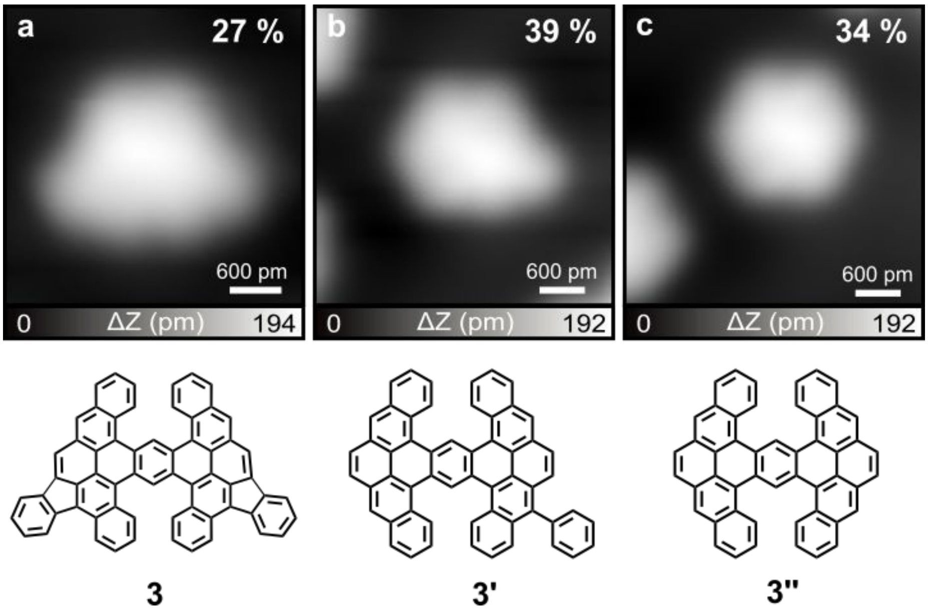

Figure S9. (a, b, c) Close-up views of STM topography for three main products (**3**, **3'** and **3''**) after annealing the sample to 250 °C. We attempted to resolve the inner structure with CO-STM but found that the molecules were accidentally moved by the tip as the same as the observation of **3**. Along with their chemical structures assumed from the planarized structures in Figure S8 and the possible reaction pathway in Scheme S1. Statistical analyses are also labeled. Measurement parameters: $V = 200$ mV and $I = 5$ pA in (a, c).

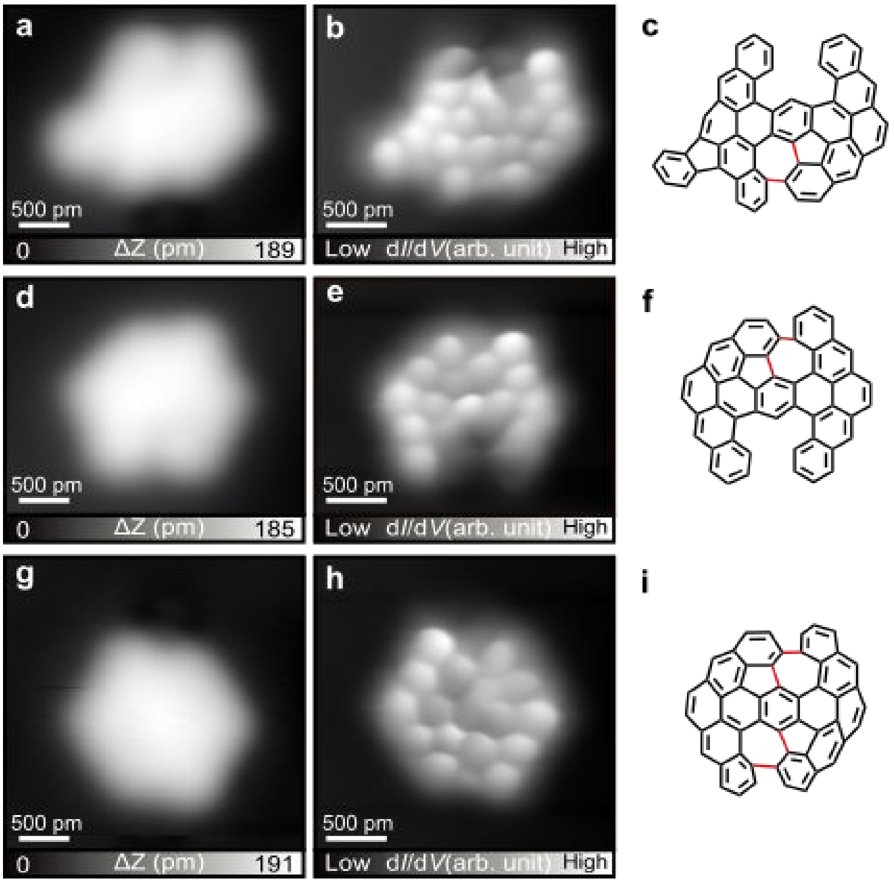

Figure S10. (a,d,g) STM topography of other planar products after annealing the sample to 350 °C, (b,e,h) the corresponding BR-STM images and (c,f,i) chemical structures. Measurement parameters: V = 200 mV and I = 10 pA in (a, d, g). V = 1 mV in (b, e, h)

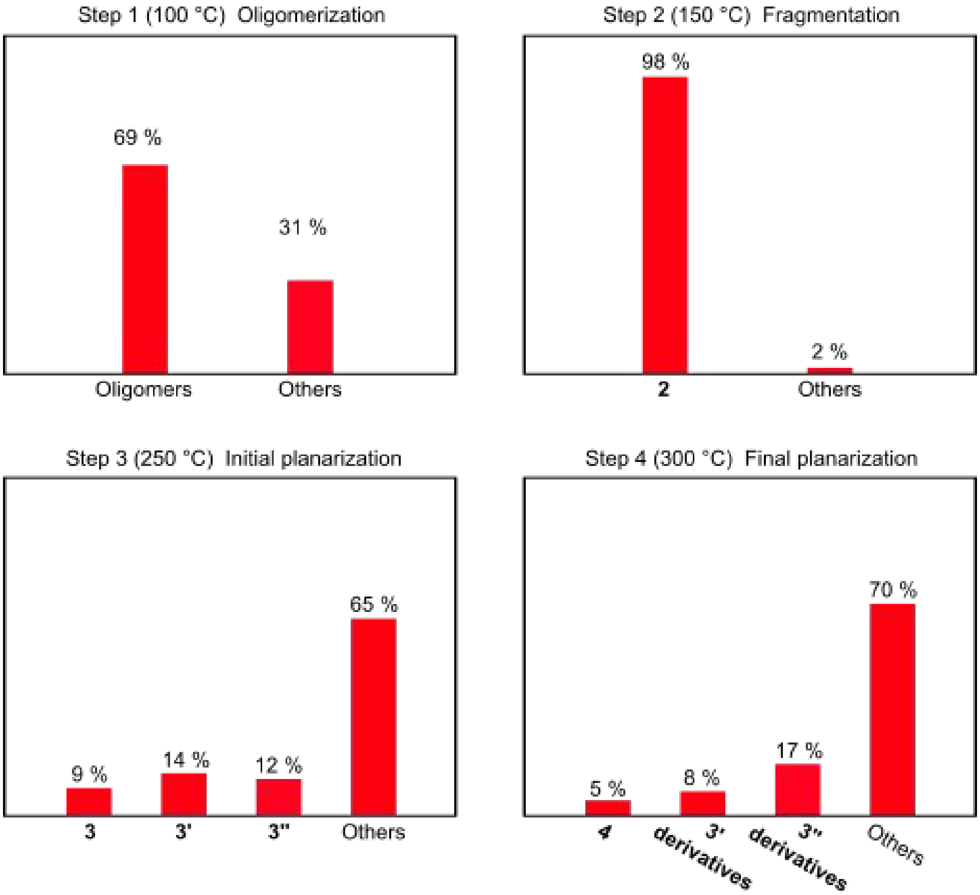

Figure S11. Statistics of the outcome molecules for each step.

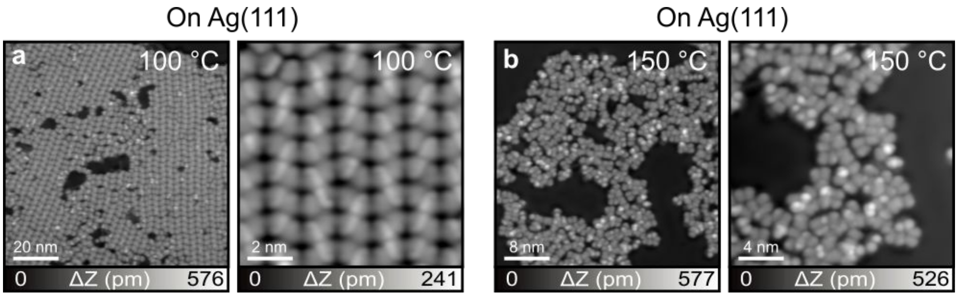

Figure S12. Formation and fragmentation of the oligomers on Ag(111). (a) Large-scale (left) and close-up (right) STM topographies taken after annealing at 100 °C. The oligomer was formed. (b) Large-scale (left) and close-up (right) STM topographies taken after annealing at 150 °C. The fragmentation was observed. (c) Large-scale (left) and close-up (right) STM topographies taken after annealing at 350 °C. On Ag(111), both the formation and fragmentation of oligomers occur, consistent with observation on Au(111). However, subsequent planarization was not observed after annealing the sample at 350 °C, indicating that the energy barrier for the planarization on Ag(111) is higher than that on Au(111). Measurement parameters: Sample bias voltage V = 200 mV and I = 10 pA in (a,b), V = 800 mV and I = 5 pA in (c).

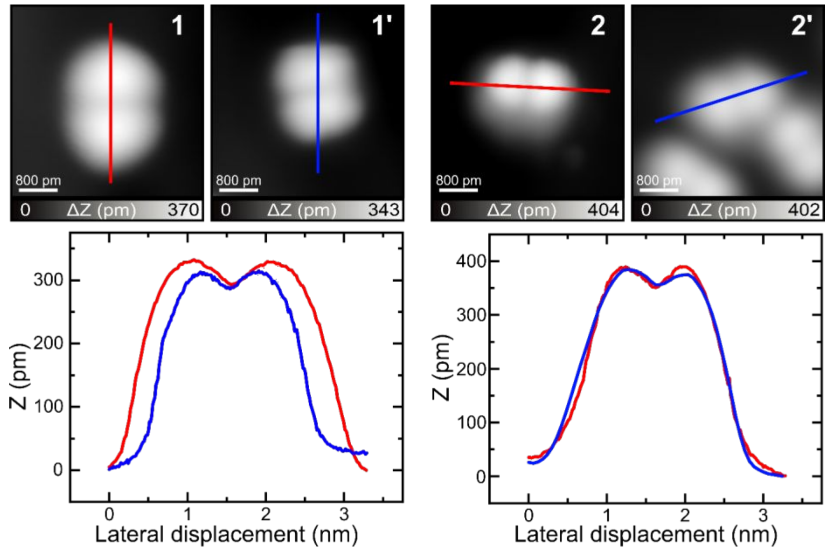

Figure S13. Comparative STM profiles for compounds **1** vs. **1'** and **2** vs. **2'**. Measurement parameters: V = 0.5 V and I = 2 pA.

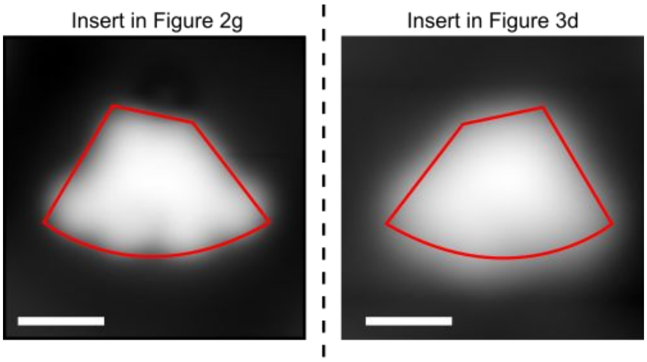

Figure S14. STM images of the products from **1** and **1'**, corresponding to the insets in Figures 2g and Figure 3d, respectively. The red-marked molecular contours highlight their mirror symmetry. The difference in contrast arises from the use of different tips: a CO-functionalized tip for the inset in Figure 2g and a metallic tip for the inset in Figure 3d.

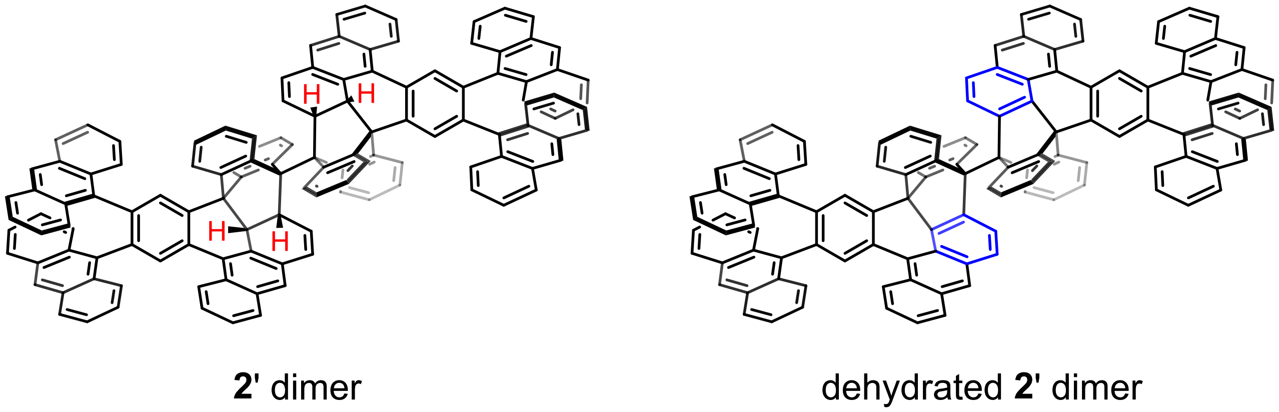

Figure S15. Structures of **2'** dimer (left) and dehydrated **2'** dimer(right).

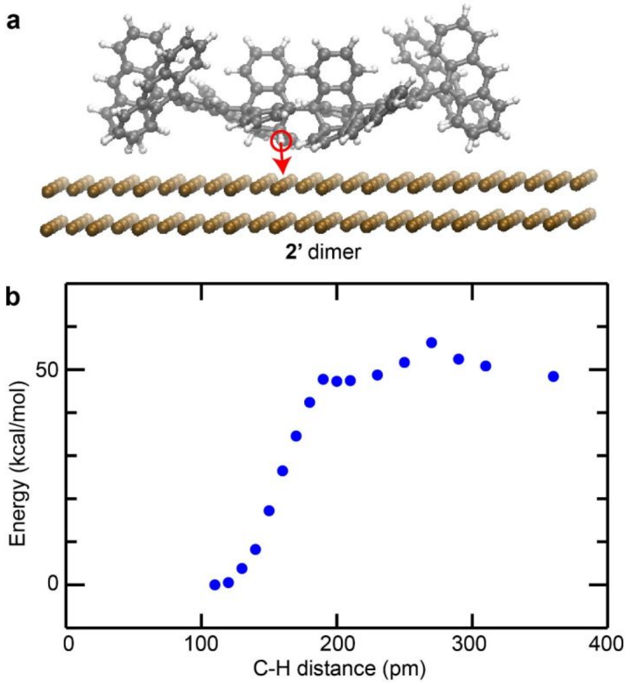

Figure S16. Dehydrogenation of **2’** investigated by DFT calculations. (a) Structures of **2’** dimer on Au(111). The red arrow indicates the pulling direction of H. (b) Potential energy surfaces of **2’** dimer on the Au(111) surface calculated by pulling the C–H distance.

^{1}H-NMR spectrum of **1** ($CDCl_3$, 700 MHz)

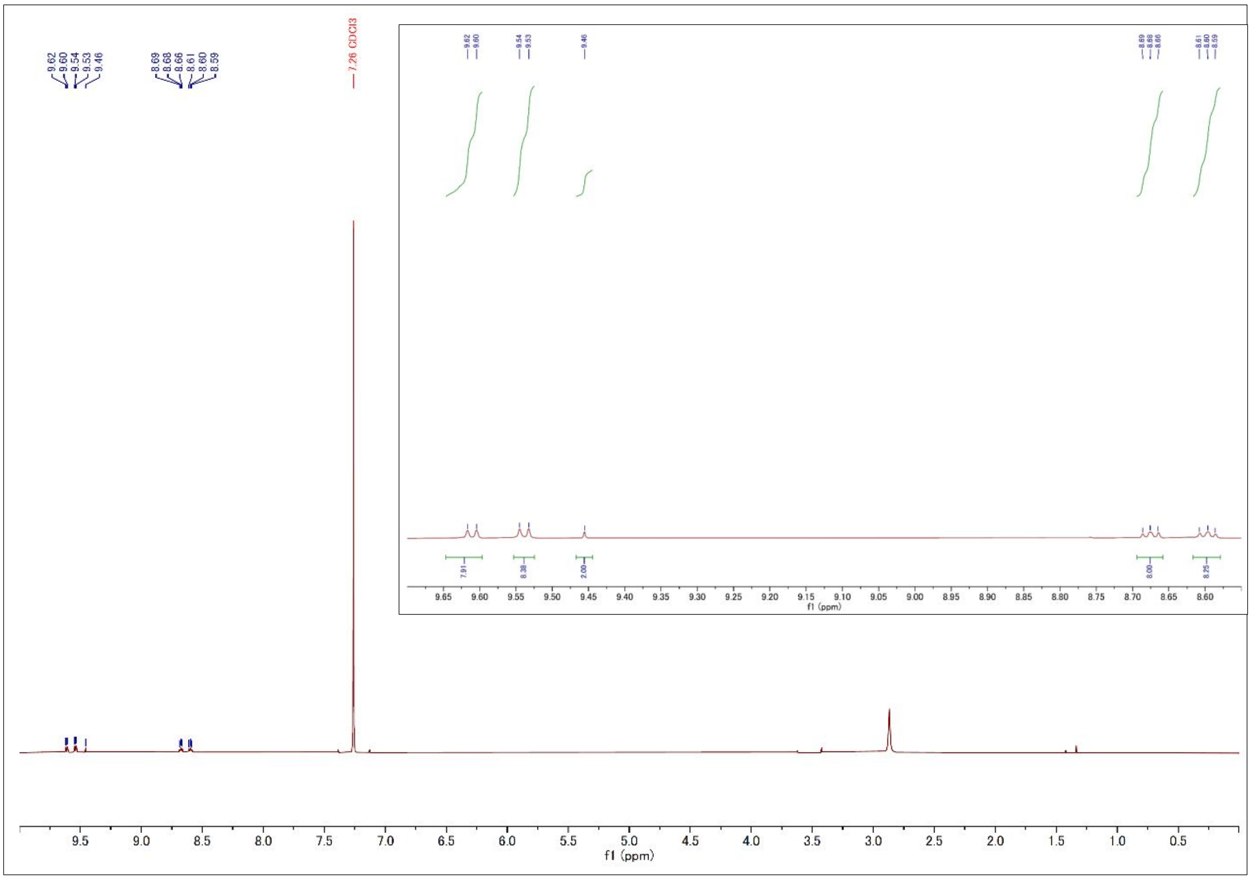

^{13}C-NMR spectrum of **1** ($CDCl_3$, 175 MHz)

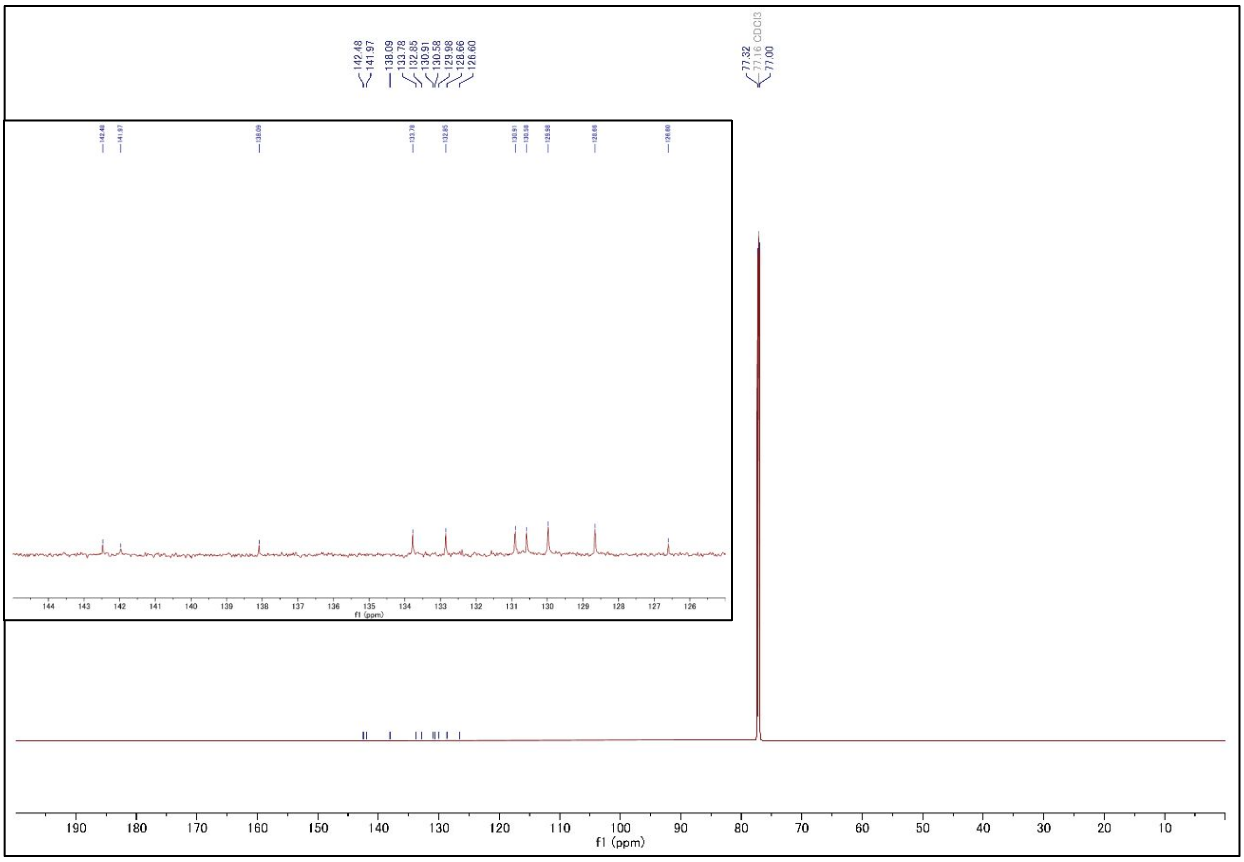

References

[S1] Nishiuchi, T.; Takeuchi, S.; Makihara, Y.; Kimura, R.; Saito, S.; Sato, H.; Kubo, T. Synthesis, properties, and intermolecular interactions in the solid states of π-congested X-shaped 1, 2, 4, 5-tetra (9-anthryl) benzenes. *Bull. Chem. Soc. Jpn*. **2022**, 95, 1591-1599.
